# Antiphotoaging Effect of 3,5-Dicaffeoyl-epi-quinic Acid against UVA-Induced Skin Damage by Protecting Human Dermal Fibroblasts In Vitro

**DOI:** 10.3390/ijms21207756

**Published:** 2020-10-20

**Authors:** Jung Hwan Oh, Fatih Karadeniz, Chang-Suk Kong, Youngwan Seo

**Affiliations:** 1 Marine Biotechnology Center for Pharmaceuticals and Foods, College of Medical and Life Sciences, Silla University, Busan 46958, Korea; wjdghks0171@naver.com (J.H.O.); karadenizf@outlook.com (F.K.); cskong@silla.ac.kr (C.-S.K.); 2 Department of Food and Nutrition, College of Medical and Life Sciences, Silla University, Busan 46958, Korea; 3 Division of Marine Bioscience, College of Ocean Science and Technology, Korea Maritime and Ocean University, Busan 49112, Korea

**Keywords:** 3,5-Dicaffeoyl-epi-quinic acid, human dermal fibroblasts, matrix metalloproteinases, photoaging, UVA

## Abstract

Cutaneous aging is divided into intrinsic and exogenous aging correspondingly contributing to the complex biological phenomenon in skin. Intrinsic aging is also termed chronological aging, which is the accumulation of inevitable changes over time and is largely genetically determined. Superimposed on this intrinsic process, exogenous aging is associated with environmental exposure, mainly to ultraviolet (UV) radiation and more commonly termed as photoaging. UV-induced skin aging induces increased expression of matrix metalloproteinases (MMPs) which in turn causes the collagen degradation. Therefore, MMP inhibitors of natural origin are regarded as a primary approach to prevent or treat photoaging. This study investigated the effects of 3,5-dicaffeoyl-epi-quinic acid (DEQA) on photoaging and elucidated its molecular mechanisms in UVA-irradiated human dermal fibroblasts (HDFs). The results show that treatment with DEQA decreases MMP-1 production and increases type I collagen production in UVA-damaged HDFs. In addition, treatment of UVA-irradiated HDFs with DEQA downregulates MMP-1, MMP-3 and MMP-9 expression via blocking MAPK-cascade-regulated AP-1 transcriptional activity in UVA-irradiated HDFs. Furthermore, DEQA relieves the UVA-mediated suppression of type I procollagen and collagen expression through stimulating TGF-β/Smad signaling, leading to activation of the Smad 2/3 and Smad 4 nuclear translocation. These results suggest that DEQA could be a potential cosmetic agent for prevention and treatment of skin photoaging.

## 1. Introduction

As the population ages, general skin deterioration of the elderly demands more attention. The various clinical, physiological and histologic changes that are common indicators of old skin are progressively involved in its vulnerability to environmental injury and disease. This deterioration requires dermatological researchers to study the biological process underlying skin aging, and the process of photoaging that is, itself, a critical clinical problem.

The considerable alteration in the appearance of the skin with aging is associated with intrinsic and exogenous factors [1]. Intrinsic aging is an insidious regressive process in living organisms over time and is related to genetic predisposition and physiological components [2,3]. Since skin is directly influenced by environmental exposure, it undergoes changes as a consequence of exogenous factors, such as solar ultraviolet (UV) radiation. UV radiation is divided into UVA (320–400 nm), UVB (280–320 nm) and UVC (200–280 nm), based on the photon wavelengths [4]. UVC is the most dangerous to skin, but it is blocked by the ozone layer. UVB can penetrate into the epidermis, resulting in DNA mutation directly. UVA penetrates more deeply into the dermis, to decompose collagen and elastin fibers via stimulating matrix metalloproteinases (MMPs). Thus, UVA is more photocytotoxic than UVB [4]. This condition is commonly known as photoaging, which accelerates the skin aging via repeated and prolonged UV radiation. Photoaging is characterized by deep coarse wrinkles, irregular pigmentation and variety of premalignant lesions [3].

UV-induced wrinkles in skin are caused by the degradation of collagen in two interdependent ways: stimulation of collagen degradation and reduction of collagen production [2]. The cellular mechanism that tightly mediates this UV-induced deterioration includes cell surface receptors, signal cascades, transcription factors and proteolytic enzymes that biosynthesize and cleave structural proteins in dermis [2]. The main mechanism behind UV exposure-initiated production of MMPs is activation of mitogen activated protein kinases (MAPK)/activator protein 1 (AP-1) pathway [5]. Studies showed that the UV-mediated MAPK signaling activates the dimeric transcriptional complex AP-1, composed of c-Fos and c-Jun, and thus regulates the expression of MMP genes [6]. MMPs are zinc-dependent endopeptidases that degrade various components of extracellular matrix (ECM) including collagen, elastin and fibronectin. To date, at least 28 types of MMPs have been reported, and they are classified into five groups, i.e., collagenases, gelatinases, stromelysins, matrilysins and membrane-type MMPs, based on their substrate specificity and structure [7]. UV radiation induces multiple MMPs, especially interstitial collagenase (MMP-1), stromelysin-1 (MMP-3) and gelatinase (MMP-9), and it causes fragmentation of collagen in skin connective tissue (dermis) [8,9,10]. Disruption of the structural and functional integrity of the collagenous-ECM is mainly responsible for the wrinkled manifestations of photodamaged skin [11]. A wealth of evidence indicates that MMP-1 initiates the degradation of type I and III collagen in photo-aged skin [6,12,13]. Once degraded by MMP-1, collagen can be further cleaved by increased levels of MMP-3 and MMP-9. Thus, regulation of MMPs is the key factor to protect the photoaging in UV-damaged skin. 

UV radiation also alters transforming growth factor β (TGF-β), a versatile cytokine that assists in regulating ECM tissue genesis and metabolism through type I procollagen biosynthesis [14]. TGF-β induces generation of ECM proteins collagen and elastin [15], and it blocks the production of proteolytic enzymes involved in the degradation of collagens, such as MMP-1 and MMP-3 [14,16]. Recent studies have demonstrated that Smad proteins are main downstream targets of stimulated TGF-β signal. TGF-β signal is propagated, downstream, through receptor-regulated Smads, such as Smads 2 and 3. TGF-β-induced Smad 2/3 form a heterodimer complex with Smad 4, which then translocates into the cell nucleus, where activation of procollagen promoter occurs. Smad 7 is known to antagonize the activation of Smad2/3 and thereby inhibit TGF-β signaling [16]. Therefore, the discovery of novel and effective MMP inhibitors or procollagen stimulators is a research hotspot in combat against photoaging.

Caffeoylquinic acid and its derivatives have been reported to show antioxidant [17], antibacterial [18], antiviral [19] and antiobesic effects [20]. Previous studies have demonstrated the protective effect of 3,5-dicafeoyl-epi-qinic acid (DEQA) in UVB-irradiated keratinocytes [21,22]. However, its applicability for treating UVA-induced photoaging in human dermal fibroblast cells (HDFs) has not been reported. Accordingly, the present study was performed to investigate the antiphotoaging effect of DEQA on both MMPs and type I procollagen pathways in UVA-irradiated HDFs and to determine its underlying mechanism.

## 2. Results

### 2.1. Cytotoxicity of DEQA Treatment and UVA Irradiation in HDFs

**Figure 1:**
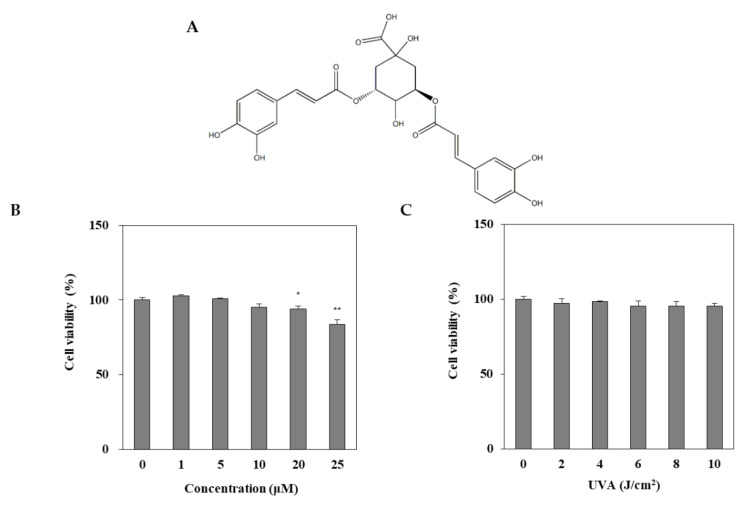
Chemical structure of 3,5-dicaffeoyl-epi-quinic acid (**A**) and its effect on viability (**B**) of human dermal fibroblasts (HDFs). HDFs were irradiated with UVA (0–10 J/cm^2^) and incubated for 24 h and the viability of the cells were measured by MTT assay (**C**). The data are presented as mean ± standard deviation of three experiments in each group. Statics represented mean ± SD of three independent experiments. * *p* < 0.05 and ** *p* < 0.01 vs. blank group.

DEQA was isolated, as reported earlier [21], and its structure is given in Figure 1A. The cytotoxic effect of DEQA on HDF was evaluated by MTT test. Treatment with DEQA (1, 5 and 20 μM) had no cytotoxic effect on HDF cells (Figure 1B). To determine the appropriate dose of UVA irradiation, the HDFs were exposed to UVA radiation in a range from 2 to 10 J/cm^2^. UVA doses up to 10 J/cm^2^ did not significantly decrease the viability of HDFs (Figure 1C). Therefore, further assay used the various concentrations (1, 5 and 20 μM) of DEQA, and the dosage of UVA irradiation was chosen as 10 J/cm^2^.

### 2.2. Effect of DEQA on the Release of MMP-1 and Collagen I in UVA-Irradiated HDFs

**Figure 2:**
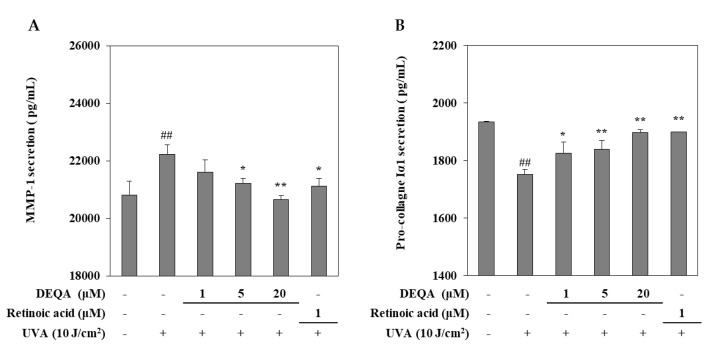
Effect of DEQA on the release of MMP-1 (**A**) and procollagen Iα1 (**B**) in UVA-irradiated HDFs. Cells were treated with given concentrations of DEQA, and then the cell-free supernatants were collected for ELISA assay. The data are presented as mean ± standard deviation of three experiments in each group. ^##^ *p* < 0.01 vs. no UVA irradiation group, * *p* < 0.05 and ** *p* < 0.01 vs. only UVA irradiation group.

The effect of DEQA on the UVA-induced changes of MMP-1 and collagen I release were measured by using an ELISA kit. UVA irradiated HDFs were cultured with DEQA and retinoic acid (positive control) for 24 h and then, the supernatants were collected to quantify the release of MMP-1 and collagen I. As shown in Figure 2, UVA exposure resulted in significantly elevated release of MMP-1 and reduced release of collagen I. UVA-induced MMP-1 release was decreased following DEQA presence in a dose-dependent manner, and the effect was significant even at low doses. Treatment with DEQA at the concentration of 20 μM more effectively inhibited the release of MMP-1 than that of retinoic acid (1 μM). Treatment with DEQA also relieved the suppression on collagen I release in UVA-irradiated HDFs. DEQA-mediated elevation of collagen I release in UVA-irradiated cells was comparable to the retinoic acid. Therefore, DEQA exhibited an effect to revert the UVA-induced changes on the release of the MMP-1 and collagen I, exhibiting possible protective effects against UVA-mediated photodegradation.

### 2.3. Effect of DEQA on MMPs and Type I Procollagen Expressions in UVA-Irradiated HDFs

**Figure 3:**
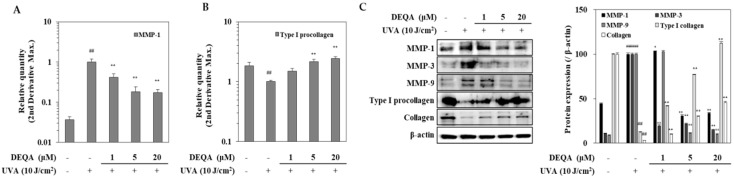
Effect of DEQA on expressions of MMPs and type I procollagen in UVA-irradiated HDFs. Cells were exposed to UVA at 10 J/cm^2^ and treated with given concentrations of DEQA for 24 h. The mRNA expression of MMP-1 (**A**) and type I procollagen (**B**) were analyzed by RT-qPCR. Protein levels of MMP-1, MMP-3, MMP-9, type I procollagen and collagen were determined by Western blotting (**C**). β-actin was used as an internal reference for RT-qPCR and Western blotting. The data are presented as mean ± standard deviation of three experiments in each group. ^##^ *p* < 0.01 vs. no UVA irradiation group, * *p* < 0.05 and ** *p* < 0.01 vs. only UVA irradiation group.

HDFs were analyzed by RT-qPCR for transcription of genes encoding MMP-1 and type I procollagen. The results in Figure 3A showed a significant increase in the MMP-1 expression in UVA-irradiated HDFs compared to the non-irradiated cells. Treatment with DEQA dramatically inhibited UVA-induced MMP-1 expression at mRNA level; MMP-1 expression level was decreased to 58.3% at 1 μM, 81.8% at 5 μM and 82.7% at 20 μM, compared with the only UVA-irradiated control group. Figure 3B showed that DEQA significantly stimulated the expression of type I procollagen in UVA-irradiated HDFs in a concentration-dependent manner; the type I procollagen expression level was elevated to 49.2% at 1 μM, 215.8% at 5 μM and 243.6% at 20 μM. Differential expression of MMP and collagen genes was further analyzed, at the protein level, by Western blotting (Figure 3C). Presence of DEQA (1, 5 and 20 μM) dose-dependently suppressed the expression of MMP-1, MMP-3 and MMP-9 in UVA-irradiated HDFs. In addition, protein levels of type I procollagen and collagen were also investigated. UVA-irradiation-induced upregulated expression of MMP-1, MMP-3 and MMP-9, and decreased expression of type I procollagen and collagen were reverted by DEQA treatment.

### 2.4. Effect of DEQA on MAPK/AP-1 Signaling in UVA-Irradiated HDFs

**Figure 4:**
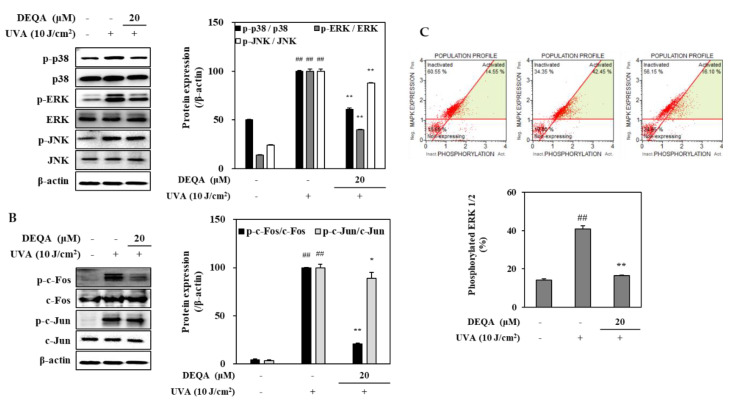
Effect of DEQA on MAPK/AP-1 signaling in UVA-irradiated HDFs. Cells were exposed to UVA at 10 J/cm^2^ and treated with 20 μM DEQA for 24 h. Phosphorylated MAPKs (p-p38, p-JNK and p-ERK) (**A**) and AP-1 activation (**B**) were detected by Western blotting. β-actin was used as an internal standard. DEQA-mediated activation of ERK1/2 in UVA-irradiated HDFs (**C**) was analyzed, using flow cytometry and MAPK activation dual detection kit. The data are presented as mean ± standard deviation of three experiments in each group. ^##^ *p* < 0.01 vs. no UVA irradiation group, * *p* < 0.05 and ** *p* < 0.01 vs. only UVA irradiation group.

To investigate whether DEQA may modulate the MMP expressions, its effects for phosphorylation of MAPKs/AP-1 were analyzed by Western blotting. The phosphorylation in MAPK signaling pathway directly stimulates the transcription factor AP-1, composed of c-Jun and c-Fos, and induces the expression of MMPs [23]. In the HDFs irradiated with UVA, three distinct MAPK cascades, namely p38, JNK and ERK, were dramatically phosphorylated soon after UVA exposure (Figure 4A). Treatment with 20 μM DEQA had an inhibitory effect on the activation of p38 and ERK signaling molecules in the UVA-irradiated HDFs (Figure 4A). As shown in Figure 4B, phosphorylation of c-Fos, part of the AP-1 transcription factor complex was suppressed by treatment with 20 μM DEQA. These findings suggested that DEQA inhibited UVA-mediated AP-1 activation by decreasing the levels of p-c-Fos. Furthermore, DEQA attenuated UVA-induced MMP expressions through suppression of the MAPK/AP-1 pathway. 

The effect of DEAQ on the inhibition of ERK1/2 phosphorylation was further investigated, using flow cytometry. In UVA-irradiated HDFs, p-ERK1/2 level was decreased by 24.4% after treatment of 20 μM DEQA (Figure 4C). These results were consistent with the protein-expression profile of phosphorylated ERK assessed by Western blot.

### 2.5. Effect of DEQA on Collagen Production in UVA-Irradiated HDFs

**Figure 5:**
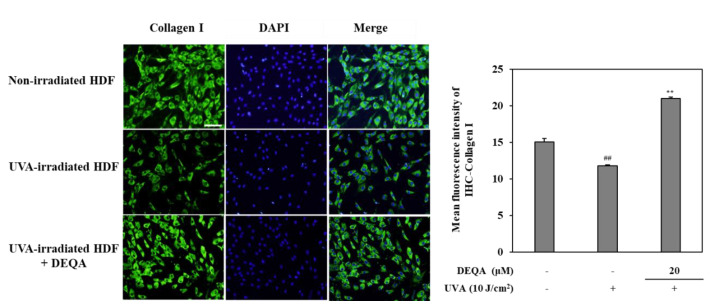
Effect of DEQA on collagen levels in the UVA-irradiated HDFs. Cells were exposed to UVA at 10 J/cm^2^ and treated with 20 μM DEQA for 24 h. Images from immunofluorescence staining of collagen I (green). Nuclei were counterstained with DAPI to reveal cell density (blue). Cellular collagen was visualized by fluorescence microscopy. Semi-quantitative analysis of collagen I staining was performed, using imageJ-software, and values were normalized against DAPI staining. ^##^ *p* < 0.01 vs. no UVA irradiation group and ** *p* < 0.01 vs. only UVA irradiation group. Scale bar: 100 μm.

In order to evaluate the protective effects of DEQA against photodegradation of collagen, an immunohistochemistry staining was performed on UVA-irradiated HDF. As shown in Figure 5, photodamaged HDFs revealed significantly lower levels of collagen compared to non-irradiated cells. Fluorescence microscopic observation verified that collagen levels in UVA-irradiated cells treated with DEAQ were significantly increased compared to the control group. These results indicated that DEQA had an attenuating effect on decreased collagen synthesis in photodamaged HDFs.

### 2.6. Effect of DEQA on TGF-β/Smad Signaling in UVA-Irradiated HDFs

**Figure 6:**
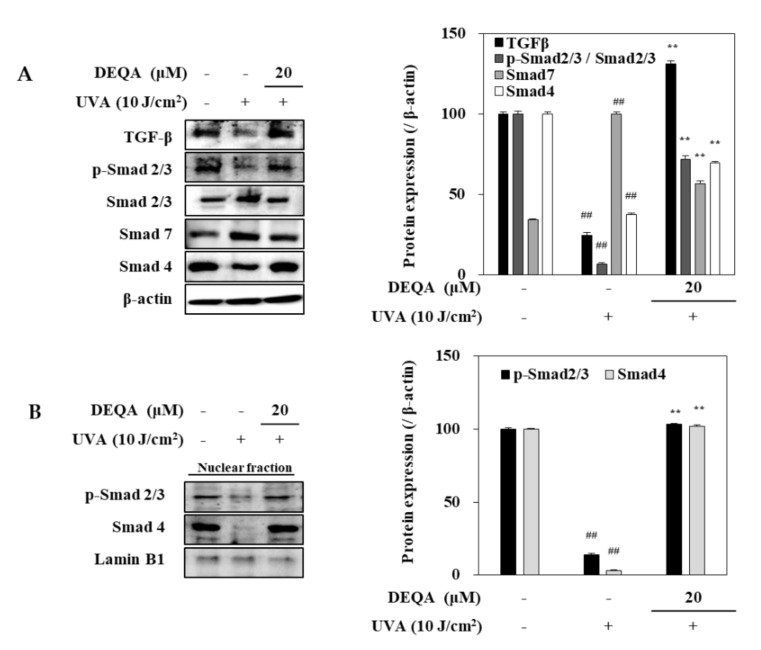
Effect of DEQA on activation of TGF-β/Smad signaling in UVA-irradiated HDFs. Cells were exposed to UVA at 10 J/cm^2^ and treated with 20 μM DEQA for 24 h. Protein expression levels of TGF-β, phosphorylated (p-) and inactive Smad 2/3, Smad 7 and Smad 4 in whole cell lysates (**A**) and phosphorylated Smad 2/3, and Smad 4 expression levels in nuclear fraction (**B**) were detected by Western blotting. β-actin was used as an internal standard in the whole lysates and Lamin B1 in the nuclear fraction. The data are presented as mean ± standard deviation of three experiments in each group. ^##^ *p* < 0.01 vs. no UVA irradiation group, and ** *p* < 0.01 vs. only UVA irradiation group.

To elucidate the underlying molecular mechanism for DEQA-mediated increase in collagen generation of UVA-irradiated HDFs, TGF-β/Smad signal transduction, a major cascade of collagen synthesis, was analyzed, using Western blotting. After UVA irradiation, protein levels of TGF-β and Smad 4 were remarkably reduced in HDFs (Figure 6A). This was also accompanied by downregulated phosphorylation of Smad 2/3 hetero complex. On the other hand, the protein level of Smad 7 which precludes Smad 2/3 phosphorylation to antagonize the TGF-β signaling was increased by UVA exposure. Treatment with DEQA enhanced the levels of TGF-β and TGF-β-induced Smad 2/3 phosphorylation compared to only UVA-irradiated HDFs. Simultaneously, the presence of DEQA reverted the UVA-induced changes on the levels of Smad 4 and Smad 7. Detrimental effects of UVA irradiation were further confirmed by reduced level of both phosphorylated Smad 2/3 and Smad 4 in the nuclear fraction of HDFs (Figure 6B). DEQA treatment attenuated the effect of UVA on nuclear p-Smad 2/3 and Smad 4 levels. Taken together, these results indicated that DEQA exerted a repairing effect on the collagen expression via TGF-β/Smad signaling cascade.

## 3. Discussion

Skin aging is a complex process influenced by both intrinsic and exogenous factors, all leading to loss of structural integrity and changes to the phenotypic and physiological characterization [2]. Typical exogenous aging, unlike intrinsic aging, is mainly affected by the cumulative effects of environmental factors, including repeated UV-radiation exposure [24]. Based on the causing agent, exogenous aging is synonymous with photoaging. Extensive UV-induced damage of the skin occurs in tandem with the deterioration of the balance between MMP levels and ECM formation [8]. Increased expression of MMP leads to degradation of collagen and other ECM proteins, and thus has a deleterious impact on skin elasticity, resulting in formation of coarse wrinkles and loss of tensile strength [25]. Therefore, the regulation of MMP production is an important strategy for preventing skin aging caused by UV-induced photodamage.

UVA irradiation decreases procollagen release from HDF cells, which may cause fragmented collagen to be found in severely photodamaged skin as a result of amplification of collagen digestion by MMPs activated in it. In the present study, it was shown that DEQA treatment dose-dependently suppressed the UVA-mediated production of MMP-1.

This result confirmed that UVA-induced reduction in procollagen secretion from HDFs was reversed by DEQA. In addition, DEQA-treated UVA-irradiated HDFs showed both decreased MMP and increased procollagen at mRNA levels. DEQA treatment of UVA-irradiated HDFs also resulted in a significant reduction of MMP-1, MMP-3 and MMP-9, and an increase of type I procollagen and collagen, at protein level, compared to only UVA-irradiated control group. Thus, the results clearly showed that DEQA attenuates UVA-induced changes in MMP and type I procollagen expressions. 

MAPK signaling cascade plays a pivotal role in MMP gene expression [26,27]. Overexposure to UV radiation can trigger the activation of MAPK pathway and its subfamilies, the p38, JNK and ERK proteins, leading to stimulation of the translocation of AP-1 [28,29,30]. The MAPK-inducible transcriptional complex AP-1, which consists of c-Fos and c-Jun proteins, translocates into the nucleus, where it induces the activation of MMP promoters. Similar to previous studies, UVA irradiation activated the phosphorylation of MAPK signaling molecules, including p-38, JNK and ERK, compared to non-irradiated HDFs. UVA-induced phosphorylation of p-38 and ERK was lowered in DEQA treated cells. This positive result suggested that DEQA attenuated UVA-induced expression of MMP by suppressing ERK and p38 MAPK phosphorylation. DEQA inhibited UVA-induced AP-1 activation by reducing c-Fos phosphorylation. Therefore, DEQA reduced UVA-induced c-Fos activation by inhibiting phosphorylation of p38 and ERK.

TGF-β cytokine and Smad proteins regulate synthesis of TGF-β dependent type I procollagen and collagen. Recent reports have demonstrated that Smad proteins are main downstream targets of TGF-β receptor kinases, acting with distinct and opposing functions [31]. In detail, TGF-β binds to the cell membrane receptor, and it leads to the phosphorylation of transcription factor, Smad 2/3. Phosphorylated Smad 2/3 assembles into heteromeric complexes with Smad 4. As a negative regulator, Smad 7 can compete with Smad 2/3 and thus reduce the activity of TGF-β/Smad cascade [31,32]. UVA irradiation can alter TGF-β pathway predominantly via decreasing the synthesis of TGF-β I and TGF-β II receptors [33,34]. The lower availability of these receptors causes reduced conjugating of TGF-β to the surface of fibroblast cells, which leads to relieved sensitivity of cells to TGF-β and downregulated phosphorylation of its downstream effector, Smad 2/3. The decreased p-Smad 2/3 caused by UVA irradiation precludes their translocation to the nucleus and consequently, their effect on the transcription factors of target genes including collagen [34]. As expected, the expression levels of TGF-β and phosphorylated Smad 2/3 were considerably increased in UVA-irradiated HDFs treated with DEQA. DEQA also decreased the expression of Smad 7 and significantly stimulated Smad 4, thus renovating TFG-β expression to the normal levels prior to UVA irradiation. These results suggested that DEQA restores type I procollagen expression by stimulating TGF-β via the effects on UVA-irradiated HDFs (i.e., Smad 2/3 phosphorylation, Smad 4 activation and Smad 7 inhibition). The TGF-β pathway was shown to be involved in the cancer-associated fibroblast (CAF) activation [35], using HDFs, and high collagen I expression was linked with normal fibroblast reprogramming in CAFs [36]. Considering the effects of DEQA on TGF- β signaling and collagen production in HDFs, further studies might promote translational applications of DEQA or its derivatives with detailed analyses of action mechanism, structure–activity relationship and effective doses. 

The present study is the first to demonstrate that DEQA reduced MMP expression and increased collagen synthesis via suppressing UVA-induced changes in HDFs. These responses might be mediated by suppression of MAPK-dependent AP-1 and increased activation of TGF-β/Smad pathways in cultured HDFs. Further studies are needed to evaluate the anti-photoaging potential of DEQA by using an appropriate in vivo model that has different genetic variations from the in vitro cellular system and is expected to exhibit a biological function attributable to them.

## 4. Materials and Methods 

### 4.1. Cell Culture and UVA Irradiation

HDFs line was purchased from Promocell (C-12302; Heidelberg, Germany). Cells were cultured in fibroblast growth medium (C-23020; Promocell) and incubated at 37 °C, in a humidified atmosphere of 5% carbon dioxide. Cells were irradiated by UVA, using a Bio-Sun UV irradiation system (Vilber Lourmat, Marine, France) fitted with a 365 nm UVA source designed for microplates. In addition, the effects of UVA irradiation were determined by the MTT assay. Following UVA (0–10 J/cm^2^) exposure and incubation of 24 h, the cell viability was observed in a dose-dependent response. The Bio-Sun UV irradiation system monitored the UV light emission based on a programmable microprocessor. The irradiation blocked automatically when the energy received coordinated the desired programmed energy (range of energy: 0 to 9999 J/cm^2^). 

### 4.2. Cytotoxicity Assay

The viability levels of HDFs were determined by an 3-(4,5-dimethylthiazol-2-yl)-2,5-diphenyl tetrazolium bromide (MTT) assay. Briefly, 1 mg/mL MTT was added to well to achieve a final concentration of 100 μg/mL after 24 h of sample treatment. Then the cells with MTT reagent were incubated in for 4 h at 37 °C. After aspiration of MTT, the formazan crystals in the cells were dissolved in dimethyl sulfoxide (DMSO) and analyzed photometrically at 540 nm, using a microplate reader (Teacan Group Ltd., Männedorf, Swiss).

### 4.3. Enzyme-Linked Immunosorbent Assay (ELISA)

To measure MMP-1 and type I procollagen, the HDFs were plated in 24-well plates at a density of 1 × 10^5^ cells/well. Cells were treated with or without DEQA (final concentrations of 1, 5 and 20 μM) immediately after UVA irradiation (10 J/cm^2^). After 24 h, the supernatants were collected and quantified for MMP-1 and type I procollagen contents by using Human Total MMP-1 DuoSet ELISA (#DY901B; R&D System Inc., Minneapolis, MN, USA) and Human Pro-Collagen I alpha 1 DuoSet ELISA (#DY6220; R&D System Inc., Minneapolis, MN, USA) kits, respectively, according to manufacturer’s instructions.

### 4.4. RNA Extraction and Quantitative Reverse Transcription Polymerase Chain Reaction (qRT-PCR) Analysis

HDFs were cultured in 6-well plates at a cell density of 1 × 10^5^ cells/well. After 24 h, cells were rinsed with phosphate buffered saline (PBS), followed by UVA irradiation (10 J/cm^2^) at 365 nm. After UVA irradiation, the cells were incubated in the presence or absence of sample for 24 h. Total RNA was isolated by using the AccuPrep^®^ Universal RNA Extraction Kit (Bioneer, Daejeon, Korea) according to the manufacturer’s instructions. cDNA was synthesized from 2 μg of total RNA with cell Script All-in-One cDNA Master Mix (CellSafe, Yongin, Korea). One step RT-qPCR was performed with Luna^®^ Universal qPCR Mix (New England Biolabs, Ipswich, MA, USA) in a thermal cycler (Dice^®^ Real Time System TP800, Takara Bio Inc., Shiga, Japan). The RT-qPCR contained 0.5 μL of cDNA, 0.5 μL of each primer (MMP-1 sense 5′-GATGTGGAGTGCCTGATGTG-3′ and antisense 5′-TGCTTGACCCTCAGAGACCT-3′, type I procollagen sense 5′-CTCGAGGTGGACACCACCCT-3′ and antisense 5′-CAGCTGGATGGCCACATCGG-3′, β-actin sense 5′-AGCCATGTACGTAGCCATCC-3′ and antisense 5′-TCCCTCTCAGCTGTGGTGGT-3′ and 10 μL (Luna universal qPCR Mix; New England Biolabs, Ipswich, MA, USA). The thermal cycling was performed in an initial denaturation step at 95 °C for 1 min, followed by 40 cycles of denaturation, at 95 °C for 15 s and 60 °C for 30 s. The gene expression was normalized to the level of β-actin, and it was quantified by using the 2^−(ΔΔCT)^ method.

### 4.5. Western Blot Analysis

The HDFs were harvested and washed three times by PBS. Total cellular protein was extracted and investigated, using immunoblotting, as previous published [29]. The nuclear fraction was extracted by NE-PER^TM^ Nuclear and cytoplasmic extraction reagents kit (Catalog No. #78835; Thermo Fisher Scientific, Waltham, MA, USA). The primary antibodies used were against MMP-1 (sc-6837; Santa Cruz Biotechnology, Santa Cruz, CA, USA), MMP-3 (sc-21732; Santa Cruz Biotechnology), MMP-9 (sc-393859; Santa Cruz Biotechnology), type I procollagen (sc-8782; Santa Cruz Biotechnology), collagen (sc-29318; Santa Cruz Biotechnology), p38 (#8690; Cell Signaling Technology, Beverly, MA, USA), p-p38 (#4511; Cell Signaling Technology), JNK (LF-PA0047; Thermo Fisher Scientific), p-JNK (sc-293136; Santa Cruz Biotechnology), ERK (#4695; Cell Signaling Technology), p-ERK (#4370; Cell Signaling Technology), TGF-β (#3711; Cell Signaling Technology), Smad2/3 (sc-133098; Santa Cruz Biotechnology), p-Smad2/3 (sc-11769; Santa Cruz Biotechnology), Smad7 (sc-101152; Santa Cruz Biotechnology), Smad4 (sc-56479; Santa Cruz Biotechnology), c-Fos (sc-7202; Santa Cruz Biotechnology), p-c-Fos (#5348s; Cell Signaling Technology), c-Jun (sc-74543; Santa Cruz Biotechnology), p-c-Jun (sc-822; Santa Cruz Biotechnology), lamin B1 (sc-374015; Santa Cruz Biotechnology) and β-actin (sc-47778; Santa Cruz Biotechnology), where secondary antibodies were anti-mouse (#7076; Cell Signaling Technology) and anti-rabbit (#7074; Cell Signaling Technology).

### 4.6. Immunohistochemistry (IHC)

HDFs were plated on to the 12-well plates which were preloaded with grass coverslips, followed by exposure to UVA irradiation (10 J/cm^2^). After UVA irradiation, HDFs were treated with or without DEQA and incubated for an additional 24 h. For general immunodetection, cells were fixed and stained, using immunofluorescence application solution kit (Catalog No. #12727; Cell signaling Technology, Danvers, MA, USA), based on manufacturer’s instructions. Briefly, cells were fixed in 4% formaldehyde for 15 min, and then immunostained with polyclonal antibody to collagen I (Catalog No. ab34710; Abcam, Cambridge, UK) at 4 °C overnight. The coverslips were washed and incubated with secondary antibody conjugated with Alexa Flour 488 (Catalog No. A-11008, Invitrogen, Carlsbad, CA, USA) for 1 h in the dark. Coverslip slides mounted in ProLong Gold Antifade reagent with DAPI (Catalog No. #8961; Cell Signaling Technology, Danvers, MA, USA) for nuclear staining. Cells were observed under a fluorescence microscope (Olympus Corp., Tokyo, Japan).

### 4.7. MAPK Activation Assay

Cells were pre-incubated in 6-well plates (1 × 10^6^ cells/well) for 24 h, and washed with PBS, followed by UVA (10 J/cm^2^) exposure. After UVA irradiation, the cells were incubated 24 h with or without different concentrations (1, 5 and 20 μM) of DEQA. To measure the total levels of ERK1/2 and phosphor-ERK1/2 proteins, central node for MAPK signaling cascade, the Muse MAPK activation Dual Detection kit (Merk Millipore, Burlington, MA, USA) was used. The kit components consisted of two types of antibodies, a phospho-specific antiphospho ERK1/2 (Thr202/Try204, Thr185/Try187) conjugated with phycoerythrin and an anti-ERK1/2 conjugated with PECy5. The percentage of the EKR1/2 activated cells in population was analyzed by the Muse^TM^ cell analyzer (Luminex Co., Austin, TX, USA), and the data were processed with Muse cell software version V1.4.0.0 (Merck KGaA, Frankfurt, Germany). 

### 4.8. Statistical Analysis

All numerical data were presented as mean ± standard deviation (*n* = 3). Statistical comparisons between the groups were performed by one-way analysis of variance (ANOVA), followed by Duncan’s test, using SPSS version 12.0 (SPSS Inc., Chicago, IL, USA).

## 5. Conclusions

In the current study, the antiphotoaging effect of DEQA was evaluated against UVA-induced photodamage. Results have shown that DEQA treatment attenuated the UVA-mediated increased production of MMP-1 and decreased production of type I procollagen. The presence of DEQA ameliorated MMP-1, MMP-3 and MMP-9 expression via blocking MAPK-cascade-regulated AP-1 transcriptional activity in UVA-irradiated HDFs. Moreover, DEQA relieved expressions of type I procollagen and collagen through reverting UVA-mediated suppression of TGF-β/Smad signaling, leading to activation of the Smad 2/3 and Smad 4 nuclear translocation. Therefore, these findings lead to supposition that DEQA could be a potential cosmetic agent for prevention and treatment of skin photoaging.
